# Progress in Medicine

**Published:** 1895-06-15

**Authors:** 


### Progress in Medicine.

### DISEASES OF CHILDREN.

(*Continued from page* 168.)

**Intestinal Diseases.**—Dr. Monti[9] gives some useful hints as to the diagnosis and localisation of intestinal obstruction. (1) Obstruction in the lower part of the colon is manifested by colic, which at first is referred to the neighbourhood of the part affected; vomiting is delayed for some hours or days, gaseous distension begins in the colon, and collapse sets in late. (2) Obstruction in the lower part of the ileum is attended with colic, starting in the right iliac region; vomiting and collapse are early symptoms; tympanites is most marked at first in the umbilical and hypogastric regions; and several motions may be passed even after the onset of complete obstruction. (3) Obstruction in the jejunum or duodenum is characterised by colicky pains about the umbilicus; by early and violent vomiting; by slight tympanites, limited to the epigastrium; and by early suppression of urine.

Dr. Jules Para[10] has found large doses of water useful in the treatment of *cholera infantum*, as recommended by Luton and Rémy. All food is interdicted for a certain number of hours, and some feebly alkalised and sparkling water is given, at first in small, and then in larger quantities, as toleration is established. He finds that when the fluid is absorbed into the blood the circulation improves and the alarming symptoms cease. Subsequently, well-diluted, sterilised milk is administered with caution.

At a discussion on the subject of intususception, Dr. Robert T. Morris,[11] showed some experiments bearing on the etiology of this affection. He touched the ileum of a rabbit with a piece of carbonate of sodium; a sudden spasm of the circular muscular fibres of the bowel was produced, and in thirty seconds there was an intussusception some two inches long, which was limited only by the crowding of the mesentery into the bowel. He suggested that, as ptomaines in some children produce convulsions, so in others they may produce local spasm and the development of an intussusception, as in the experiment.

**Nervous Diseases.**—Dr. Andrew Macphail,[12] of Montreal, has given a short account of an epidemic of paralysis, affecting children for the most part, but also including a few adults. This occurred in the State of Vermont during the months of June, July, and August, 1894, and the total number of patients affected was 120. Premonitory symptoms appeared in some cases, namely, headache, sickness, vomiting, pyrexia, &c., but in others the onset of paralysis affecting one or more limbs was the first symptom observed. The paralysis was essentially motor in type, and the only sensory alteration noted was hyperæsthesia. The statistics given show a mortality of 13 per cent., recovery in 25 per cent. of the cases, improvement in 30 per cent., and no improvement in 32 per cent. Further details are to be supplied when the pathological and bacteriological investigations have been completed.

A case of infantile hemiplegia successfully treated by trephining is recorded by Dr. Edward B. Angell.[13] The patient was a boy aged six years, who had suffered from birth from paralysis of the right arm and leg. His moral condition was bad, the child being destructive, quarrelsome, and addicted to the use of bad language. Control over the functions of the bladder and rectum was lost, and he suffered from epileptic seizures daily. At the operation, after the skull was trephined, the dura mater bulged slightly, and the existence of a cyst was proved by the withdrawal of a drachm of reddish fluid. The cyst was not evacuated, but the trephine opening was enlarged until it extended beyond the boundaries of the cyst, and the scalp-wound was then stitched up. It was considered advisable to leave the cyst undisturbed; firstly, because of the increased risk in the operation from opening the dura mater; and, secondly, because the symptoms were apparently due to pressure which had been relieved. The result was satisfactory; walking power improved, the mental and moral condition of the patient changed for the better, and the fits soon ceased entirely.

Dr. A. Breton[14] has described two varieties of mental complications in chorea. In the first class he places alterations of moral sensibility, of character, and of intelligence, want of attention and loss of memory; and, in the second, night-terrors, hallucinations, and "folie choreique." The last-named may take the form of simple or acute mania, or melancholia, with suicidal tendencies. Recovery from these psychical alterations usually accompanies the cessation of the motor disturbances, but in some cases no improvement takes place, and this is more especially the case when there is a family history of nervous disease.

**Biliary Cirrhosis in Children.**—At the Indian Medical Congress papers were read on this subject by Dr. J. N. Ghose and Dr. E. Mackenzie.[15] This would appear to be a new disease, or at least one regarding which little has been known. It seldom occurs in children over three years of age, and those under one year are particularly liable. As regards the etiology, no explanation is forthcoming, although the writers have made careful inquiry into the questions of constitutional disease, diet, locality, class, and caste of the parents. Dr. Ghose is inclined to trace the affection to improper feeding and mal-assimilation. It is rarely seen amongst the children of European parents. In one family fourteen children died in succession from this malady. It seems to run its course in from three to twelve months, although a fatal issue has occurred within a fortnight of the onset of the first symptoms. Out of 400 cases only six recovered. The early symptoms are loss of appetite, nausea and sickness, sallow complexion, thirst, constipation, and slight pyrexia, but the liver may become considerably enlarged before any of these are manifested. When the patient is first seen the liver is usually found to extend to the level of the umbilicus or even of the iliac crest. Jaundice, ascites, and anasarca develop rapidly, and death follows from hæmatemesis, or coma. The liver is found *post-mortem* to be stained yellow, enlarged, and indurated owing to an excessive growth of fibrous tissue between the lobules, around the bile-ducts, and in the lobules themselves. The hepatic cells are atrophied from pressure. Serous effusion takes place into the peritoneal, pleural, and pericardial cavities, the kidneys are soft and congested, and the spleen is enlarged and friable. Both writers state that so far no treatment has been of the slightest use in checking the progress of the disease.

[9] Allg. Wien. Med. Zeit., 1894, Nos. 35, 36, and Ep. B.M.J., Nov. 3, 1894. [10] Rev. Mens. des Malad. de l'Enf., Sep., 1894, and Am. Jour. of Med. Sci., Dec., 1894. [11] Am. Jour. of Obstet., Nov., 1894, p. 744. [12] B.M.J., Dec. 1, 1894. [13] Jour. of Nerv. and Ment. Dis., Oct., 1894. [14] L'Union Med., Oct. 27, 1894, and the Lancet, 1894, II., p. 1173. [15] The Lancet, Feb. 2, 1895.

